# Eight-Year Follow-up of a Girl with McCune-Albright Syndrome

**DOI:** 10.4274/jcrpe.v3i1.09

**Published:** 2011-02-23

**Authors:** Zehra Aycan, Aşan Önder, Semra Çetinkaya

**Affiliations:** 1 Dr. Sami Ulus Obstetrics and Gynecology, Children's Health and Disease Training and Research Hospital, Ankara, Turkey; +90 312 305 65 08 70 +90 312 347 23 30 asanonder@yahoo.com Dr. Sami Ulus Obstetrics and Gynecology, Children's Health and Disease Training and Research Hospital, Ankara, Turkey

**Keywords:** McCune-Albright syndrome, bisphosphonate, aromatase inhibitor, follow-up

## Abstract

McCune-Albright syndrome (MAS) is characterized by the triad of fibrous dysplasia (FD), cafe-au-lait spots and precocious puberty (PP). We report a 14-year-old girl with MAS who has been followed-up for 8 years. She was referred for multiple fractures and vaginal bleeding at age 5.9 years. She had peripheral PP, FD, and osteoporosis and was diagnosed as MAS. The patient was treated with aromatase inhibitors and bisphosphonates. She had no menses during aromatase inhibitor treatment. Her growth rate and bone maturation were in normal ranges while on treatment. She had one new fracture on the seventh year of follow- up in spite of bisphosphonate treatment.

**Conflict of interest:**None declared.

## INTRODUCTION

McCune-Albright syndrome (MAS) is characterized by fibrous dysplasia (FD), café-au-lait skin spots and precocious puberty (PP). The syndrome may be associated with hyperfunction of other endocrine glands, such as growth hormone excess, hyperprolactinoma, hyperthyroidism and Cushing’s syndrome (1,2,3). MAS is a rare disorder and its prevalence is estimated to be between 1/100 000 and 1/1 000 000 (4). Activating somatic mutations of GNAS gene located on chromosome 20q13 encoding the a-subunit of the regulatory G_s_a protein are responsible for the entity (5,6).

PP is the most common endocrinopathy seen in MAS and occurs in 64-79% of girls and 15% of boys (3). Aromatase inhibitors (testolactone, fadrazole, anastrozole, letrozole), tamoxifen (selective estrogen reseptor modulator), gonadotropin-releasing hormone (GnRH) analogues and surgery are the different treatment strategies employed in the treatment of PP due to MAP (7). 

FD is reported in 46-98% of MAS patients. FD presents as an isolated lesion involving a single site in one third of these patients. Calcium and vitamin D supplementation, intravenous pamidronate therapy and surgical methods are used in the management of FD (8,9). The prevalence of café-au-lait skin spots varies between 53.1% and 92.5% in MAS (3).

In this report, we present the long-term follow-up findings of a patient diagnosed as MAS. 

## CASE REPORTS

A 5.9-year-old girl was referred to our Pediatric Endocrinology clinic for premature menarche and three fracture episodes that have occurred in the past year. On physical examination, she was noted to have thelarche (Tanner stage 2) and multiple café-au-lait skin spots. Her height was 116 cm (-0.35 SD). GnRH stimulation test revealed suppressed gonadotropin levels (peak LH: 0.4 mIU/mL, peak FSH:0.92 mIU/mL) and an elevated estradiol (E2) level (29.8 pg/mL). Her bone age was 8 years, assessed by the Greulich-Pyle method. On pelvic ultrasound, uterine size was 30.3x12.3x11.9 mm, the dimensions of the right ovary were 18.5 x15 mm and those of the left ovary 16.8 x 15.3 mm. A follicle cyst 11 mm in diameter was detected in the right ovary without any other pathology. The serum level of thyrotropin (TSH) was 0.09 mIU/mL (normal: 0.6-5.5 mIU/mL), of free thyroxine (T4) 1.41 ng/dL (normal: 0.8- 1.9 ng/dL) and that of free triiodothyronine (T3) was 4.57 pg/mL (normal: 2-7.6 pg/mL). TSH levels were suppressed after thyrotropin-releasing hormone (TRH) administration. Thyroid autoantibodies were negative. Multinodular goiter was present on thyroid ultrasound. Bone scintigraphy revealed increased activity concordant with FD in the craniofacial bones, in the right femur and right humerus. She had a lumbar spine Z-score of -2.7 on dual-energy X-ray absorptiometry (DXA) scan. Serum cortisol, prolactin, growth hormone, insulin-like growth factor-1 (IGF-1) and IGF-binding protein-3 (IGFBP-3) levels were in normal ranges. Based on the coexistence of PP, FD and café-au-lait skin spots, the patient was diagnosed as MAS. 

Testolactone (200 mg/day) therapy was initiated. After the first year of treatment, testolactone was discontinued. Anastrazole was started and used for four years. On this treatment, serum E2 levels ranged between 29 and 59.3 pg/mL, LH - 0.15 and 0.2 mIU/mL, and FSH between 0.1 and 2 mIU/mL. Pelvic ultrasonography revealed uterine volume between 6.2 and 10.2 ml with persisting follicular cysts of 23-32 mm in diameter. Growth rate was 6 cm/year during this period. Anastrozole treatment was discontinued when the patient reached 11 years of age. At this time, she was at pubertal stage 3, with a bone age of 12 years. She started to have spontaneous menses six months after discontinuation of anastrazole therapy. Her menstruation periods were irregular at the beginning, but she has had regular menses in the past three years. She was 153.8 cm (-0.9 SD), 50.1 kg (-0.1 SD) and had achieved Tanner stage B5 in her recent control when she was 14 years old. 

As we investigated for other endocrinopathies, we found that she had subclinic hyperthyrodism; she was followed up with beta- blocker therapy during the first four years. Subclinic hyperthroidism status had come to an end on the fifth year and beta-blocker treatment was terminated. Subclinical hyperthyroidism status resolved in the fifth year and beta-blocker treatment was terminated. Her ultrasound scans were concordant with multinodular goiter and the fine-needle aspiration biopsy specimens showed benign cytology. Our patient was in euthyroid status without any treatment during the last four years. Thyroid ultrasound and fine-needle aspiration biopsy were planned to be annually performed. During the follow-up period, no other endocrinopathies (e.g. hypercortisolism, hyperprolactinoma or growth hormone excess) that may accompany MAS were noted. There was no pathology on the MR images of the sella either. 

For FD and osteoporosis, three cycles of pamidronate (1mg/kg/cycle every four months) were given in the first year. Then, the therapy was continued with oral etidronate and vitamin D plus calcium supplementation. In the third year after the diagnosis, she had a lumbar spine Z-score of -0.1 on DXA scan and experienced no fractures during this period. Treatment was therefore discontinued. However, because of abnormal findings on plain X-ray films, intravenous pamidronate (2 mg/kg/cycle every six months) and calcium, as well as vitamin D treatment were restarted on the sixth year of follow-up when she was 11 years old. Despite this treatment, the patient had one new fracture in the seventh year of follow-up when she was under pamidronate therapy and her bone mineral density (BMD) L1-L4 Z-score was 0.29. Serum calcium, phosphate, alkaline phosphatase and parathormone levels were in normal ranges during follow-up and there was no phosphate wasting or hyperparathyrodism.

## DISCUSSION

MAS is defined as the triad of FD, café-au-lait skin spots and PP. Other pathologies such as hyperthyroidism, growth hormone excess, renal phosphate wasting (with or without rickets/osteomalacia), Cushing’s syndrome, as well as liver, parathyroid, pancreas and heart involvement may accompany MAS (2). Activating mutations in exon 8 of GNAS gene is the cause of this disorder. These mutations lead to impaired GTPase activity and result in cAMP increase and unregulated hormone production. Mutation formation is a post-zygotic somatic event. Hence, there is a mosaic distribution of cells containing the mutation and there is a great variability in tissue involvement and disease severity (1,6,10).

PP is the most common endocrinopathy seen in MAS. PP affects girls more often than boys and frequently, vaginal bleeding is the initial sign. Autonomic activation of ovarian tissue leads to ovarian follicular cysts and estrogen hypersecretion. This status is defined as peripheral PP since hypothalamic-pituitary-gonadal axis is inactive. However, progression to central PP may occur at follow-up (7,11). Historically, ketoconazole and medroxyprogesterone were used in the treatment of PP. Medroxyprogesterone does not seem to have an effect on accelerated skeletal maturation, but may stop the vaginal bleeding. Treatment approaches include anti-estrogens (aromatase inhibitors-testolactone, fadrozole, anastrozole, letrozole), estrogen receptor blockers (tamoxifen), GnRH analogues (if there is a progression to central PP) and surgical methods (unilateral oophorectomy, cystectomy) (7). Nunez et al (12) indicated that fadrazole is not effective and must not be used as it may cause adrenal insufficiency. Feuillan et al (13) reported that testolactone can be used in the treatment of PP since it leads to a decrease in ovarian volume and estrogen levels. In another study, these same authors found that letrazole is an effective agent in the treatment of PP in MAS (14). 

Our patient did not have menses under aromatase inhibitor treatment and peripheral PP did not progress to central PP. There was no significant increase in ovarian volumes when she was receiving testolactone. However, the follicle cysts in the right ovary persisted on serial sonographic examinations. Although anastrazole may be ineffective (15), it was found to be beneficial in our patient. Although it has been reported that the pituitary-gonadal axis may frequently be suppressed in late adolescence or adulthood due to persisting ovarian hyperfunction and hyperestrogenism seen in these subjects (11), our patient had regular menses and her gonadotropin levels were within the pubertal reference ranges. Most of the reported MAS cases have oligo-amenorrhea or hypermenorrhea and rarely, regular menstrual periods. There may be infertility problems secondary to ovarian hyperfunction and this underlines the need for close follow-up of ovarian function in women with MAS. 

It has been reported that thyroid involvement occurs in approximately 30% of MAS patients (16). Hyperthyroidism in MAS is associated with an increase in the free T3/ free T4 ratio. Free T4 levels are mildly elevated or normal. Autoimmunity does not accompany this status (16). Multinodular goiter may be detected by ultrasonography. Our case also received beta-blocker treatment for subclinical hyperthyroidism during the first four years after the diagnosis.

It has been shown that bisphosphonate treatment decreases fracture rate and pain in FD (17). FD lesions have high interleukin (IL)-6 levels leading to increased osteolytic activity. Bisphosponate treatment prevents this by inhibiting IL-6 (18). The levels of osteoblastic/osteolytic markers decrease secondary to bisphosphonate therapy (8). Nevertheless, it has been reported that calcium and vitamin D supplementation is the first step in FD treatment. Bone pain is a clear indication for bisphosphonate treatment in childhood (8,9). We prescribed bisphosphonates to our patient as she complained from fractures, bone pain and had a low BMD at diagnosis. On therapy, she did not suffer from bone pain and her BMD increased. We did not observe any side effects secondary to this treatment. However, our patient had a new fracture under bisphosphonate treatment in spite of the increase in BMD. 

MAS is a rare disease and there is no consensus or sufficient experience regarding the time to terminate PP treatment and about the problems that may occur during the late adolescent or adult period. 
